# More creative through positive mood? Not everyone!

**DOI:** 10.3389/fnhum.2012.00319

**Published:** 2012-11-26

**Authors:** S. Akbari Chermahini, Bernhard Hommel

**Affiliations:** Institute for Psychological Research and Leiden Institute for Brain and Cognition, Leiden UniversityLeiden, Netherlands

**Keywords:** divergent thinking, creativity, dopamine, emotion, eye blink rate

## Abstract

It is commonly assumed that positive mood improves human creativity and that the neurotransmitter dopamine might mediate this association. However, given the non-linear relation between dopamine and flexibility in divergent thinking (Akbari Chermahini and Hommel, 2010), the impact of mood on divergent kinds of creativity might depend on a given individual's tonic dopamine level. We tested this possibility in adults by assessing mood, performance in a divergent thinking task [the Alternate Uses Task (AUT)], and eye blink rates (EBRs), a well-established clinical marker of the individual dopamine level, before and after positive mood or negative mood induction. As expected, the association between flexibility in divergent thinking performance and EBR followed an inverted U-shape function (with best performance for medium levels), positive mood induction raised EBRs and only individuals with below-median EBRs, but not those with above-median EBRs, benefited from positive mood. These observations provide support for dopamine-based approaches to the impact of mood on creativity and challenge the generality of the widely held view that positive mood facilitates creativity.

Creativity is arguably the most potent human resource both for the advancement of mankind in general and people's individual progress and success in daily life in particular. And yet, the cognitive and neural mechanisms underlying creative behavior are poorly understood. Researchers agree that at least some forms of creativity vary with mood and two recent meta-analyses have concluded that performance in tasks tapping divergent (brainstorm-like) thinking can be reliably improved by inducing positive mood (Baas et al., 2008; Davis, 2009). This conclusion fits with earlier considerations of Isen (1987), who claimed that positive affect (PA) impacts cognitive processing by (1) increasing the number of cognitive elements available for association; (2) defocusing attention so to increase the breadth of those elements treated as relevant to the problem; and (3) increasing cognitive flexibility.

Exactly how positive mood manages to improve creativity is not clear yet, but in approaches that tackle this issue the neurotransmitter dopamine (possibly in concert with other neurotransmitter systems: Cools et al., 2008) plays a major role. Notably, Ashby et al. (1999) have pointed out that phasic changes in dopamine levels, mood changes, and changes in creativity (especially in cognitive flexibility) may be strongly interrelated. Their approach is inspired by insights into the neurobiology of reward, the encounter of which has been shown to induce both PA and phasic increases of dopamine levels (e.g., Beninger, 1991; Bozarth, 1991; Phillips et al., 1992; Schultz, 1992). Accordingly, Ashby and colleagues ( 1999) suggest that improved mood states are accompanied by phasic increases in dopaminergic supply provided by frontal and striatal pathways. These phasic increases might facilitate switching from one task set or item to another, thereby increasing cognitive flexibility in creativity tasks. This scenario is consistent with results from neural-network modeling (Cohen and Servan-Schreiber, 1992; Ashby et al., 1999) and the observation that divergent thinking performance interacts with individual differences in the DRD2 TAQ IA gene—which affects receptor density in the striatal dopaminergic pathway (Reuter et al., 2006). Moreover, the personality trait of “seek,” which has been claimed to rely on dopaminergic pathways (Panksepp, 1998), has been reported to be positively related to creativity (Reuter et al., 2005).

To assess the connection between creativity and dopamine, Akbari Chermahini and Hommel (2010) related individual performance in a divergent thinking task to spontaneous eye-blink rates (EBRs), an indirect but well-established clinical marker of the individual dopamine level (Karson, 1983; Blin et al., 1990; Kleven and Koek, 1996). Flexibility in divergent thinking (or cognitive flexibility for short) did in fact covary with EBR but the function relating these two measures was non-linear and followed an inverted U-shape^1^. As indicated in Figure 1, an idealized function modeled after Akbari Chermahini and Hommel's findings, individuals with medium EBR were performing better than individuals with low or higher rates did (individuals with particularly high rates were not tested in this study,^2^). If we take EBRs as a marker of the current dopamine level (presumably integrating tonic and phasic levels), this has a number of rather serious implications that we set out to test in the present study.

**Figure 1:**
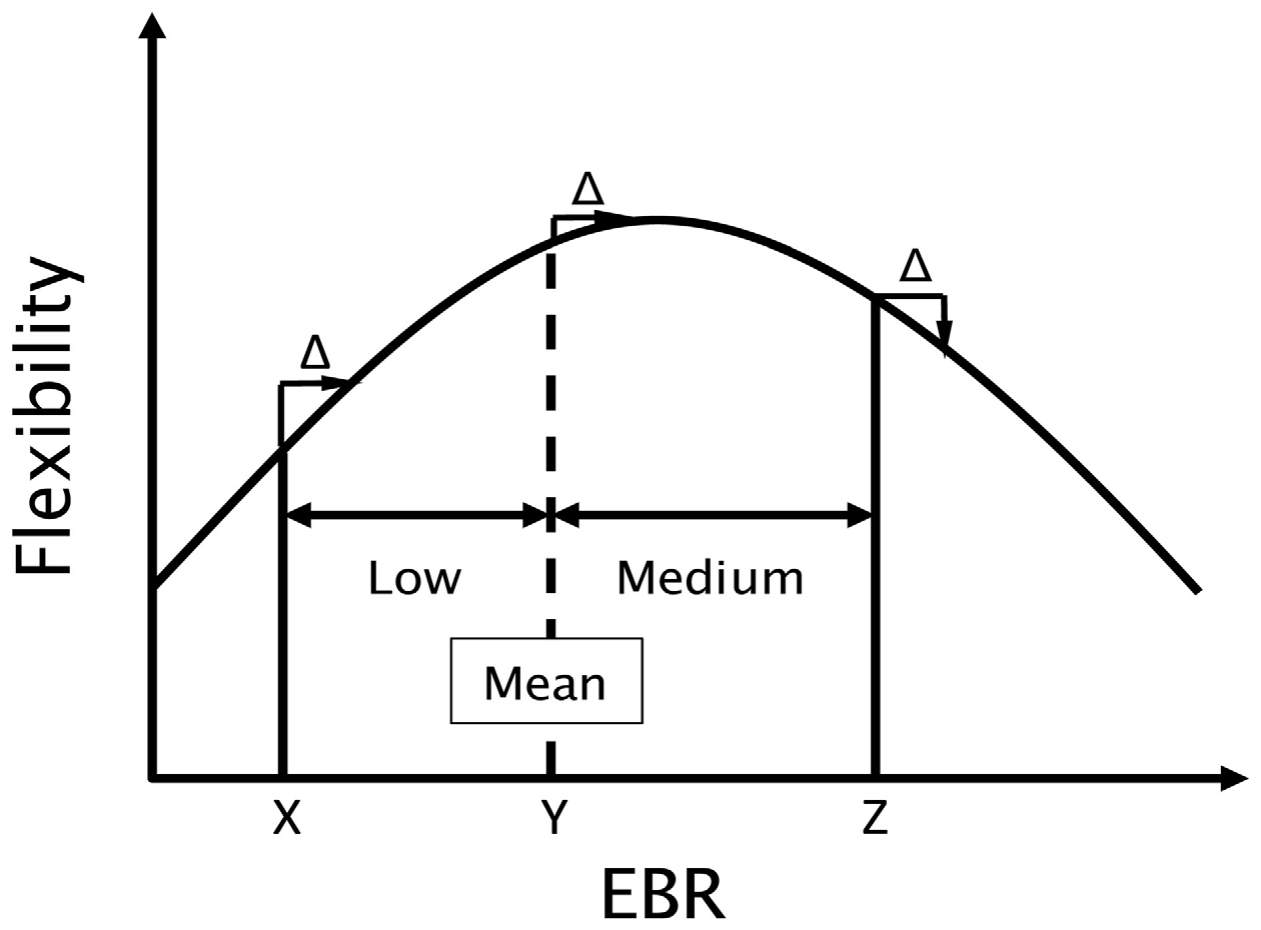
**Hypothetical function (modeled after Akbari Chermahini and Hommel, 2010) relating flexibility in divergent thinking to eye blink rate (EBR), an estimate of the individual dopamine level.** The estimate of the group mean is taken from Akbari Chermahini and Hommel (2010). Note that an increase in dopamine (EBR) of Δ would strongly increase performance of the hypothetical individual “X,” only mildly improve performance of “Y,” and impair performance of “Z.”

First, it suggests that EBR can be used to monitor the impact of mood manipulations. If it is the case that inducing positive mood increases the current dopamine level and if EBR indeed reflects this level, we should be able to demonstrate that inducing positive mood leads to an increase in EBR. Whether we should expect the induction of negative mood to decrease EBR was less clear. On the one hand, there is evidence that dopamine neurons are activated by events that are more rewarding than predicted and depressed by events that less rewarding than predicted (Schultz, 1992), suggesting that positive and negative mood might increase and decrease EBR in a symmetric fashion. On the other hand, however, numerous findings suggest that negative mood is not just the opposite of positive mood (e.g., Baas et al., 2008), which fits with the increasing evidence that while positive mood is heavily affected by dopamine, negative mood is more strongly linked to serotonin (e.g., Cools et al., 2008; Dayan and Huys, 2008). Considering this possibility, EBR and cognitive flexibility might be more impacted by positive than by negative mood.

Second, if we take both mood and EBR changes as reflections of phasic dopaminergic changes, the *amount* of mood and EBR changes should be systematically related to the *degree* of change in cognitive flexibility. That is, elevated mood and increased EBRs should be associated with improved flexibility, whereas negative-going mood might rather be associated with decreased EBRs and impaired flexibility.

Third, Akbari Chermahini and Hommel (2010) observation that cognitive flexibility relates to EBR in an inverted U-shaped fashion suggests that the impact of increasing (or decreasing) the individual dopamine level on flexibility should depend on the basic level of the corresponding individual. Consider, for instance, an individual with a relatively low level of dopamine, as the hypothetical person “X” in Figure 1. In view of Akbari Chermahini and Hommel's findings, this individual would be expected to perform rather poorly with respect to cognitive flexibility. Inducing positive mood would be expected to increase the dopamine level by some hypothetical amount Δ and thereby move this individual more toward the central zone of the performance function, which is associated with the best performance. Hence, positive mood induction should be beneficial for individuals with low EBR. Positive mood should also be beneficial for individuals with higher EBRs, as long as the rate falls on the ascending flank of the function. Accordingly, the hypothetical person “Y” would show a benefit, which however would be smaller than that shown by “X.” However, for individuals with even higher EBR, such as person “Z” in Figure 1, positive mood should no longer improve flexibility but have no effect or even impair performance. Hence, we would expect that people with a low pre-experimental EBR would be expected to benefit from positive mood more than people with medium or relatively high pre-experimental EBRs do.

We assessed these three hypotheses in the following way: Participants were first tested on general, pre-experimental mood (for both their general and their current mood state), on performance in divergent thinking, and on their pre-experimental EBR. Then two subgroups of participants underwent a positive mood and negative mood induction, respectively, before again being tested on mood, divergent thinking, and EBR.

## Methods

Eighty-one native Dutch students of Leiden University volunteered in exchange for course credit or pay. The study consisted of three phases, which together took 45 min to complete. First, all participants filled out an inventory assessing their general mood Positive and Negative Affect (NA) Scales (PANAS) and a mood inventory assessing their current mood state (MI1), before performing a divergent-creativity task (Alternate Uses Task: AUT1); finally, their spontaneous EBR were measured (EBR1). In the second phase, 43 participants received a positive mood induction while 38 participants received a negative mood induction. In the third phase, another version of the mood inventory (MI2) was filled out, EBR2 was measured, and another version of the divergent thinking task was performed (AUT2). The order of the two versions of the mood inventory and the divergent thinking task was counter-balanced across participants. EBR2 was measured after mood induction while subject continually was thinking about either happy or sad memory.

### Positive and negative affect scales (PANAS)

The PANAS (Watson et al., 1988) is 20-items self-report mood scale that measures general (“how do you feel generally?”) PA and NA. It comprises of 10 positive and 10 negative adjectives rated on a Likert scale from 1 (very little or not at all) to 5 (very or extremely). We used a Dutch version of the scale with high internal consistencies for the PA (Cronbach's alpha = 0.84) and the NA (Cronbach's alpha = 0.80) subscale (c.f., Hill et al., 2005).

### Mood inventory (MI)

Instead of presenting the PANAS repeatedly (which would have invited memory biases), we used two Dutch versions of a mood inventory (developed by Phillips et al., 2002, and similar to the scale of Isen et al., 1987) to assess current mood in the first and the third phase of the experiment. Three of the five items of this inventory assess the hedonic quality of affect (Phillips et al., 2002). One version (Cronbach's alpha = 0.75) used the following adjective pairs (Dutch words are given in parentheses) to measure valence: happy–sad (*blij-verdrietig*), peaceful–anxious (*vredig-angstig*), and carefree–serious (*zorgeloos-serieus*). The second version (Cronbach's alpha = 0.85) used the pairs: positive–negative (*positief-negatief*), calm–uptight (*kalm-opgewonden*), and bright–dispirited (*helder-serieus*). Positive and negative words were presented on the left and right side of a page, respectively. Nine-point Likert scales separated the words of each pair and participants were asked to rate their current mood state (following Phillips et al., 2002). For analytical purposes the mood scores were reversed and then totaled, so that higher scores indicate more positive mood.

### Alternate uses task (AUT)

Following Guilford (1967), participants were asked to write down as many possible uses for a common household item as they can within 5 min. Two different items were used: *cup* and *pencil*. The order of the two items was balanced across participants, so that half of the participants received the *cup* item before and *pencil* after mood induction, while the other half received the opposite sequence. Responses were scored with respect to flexibility, originality, fluency, and elaboration (Guilford, 1967). However, given that flexibility is most strongly and reliably related to EBR measures (Akbari Chermahini and Hommel, 2010), we focused on the flexibility score^1^, which is derived from the number of different categories being used for each item.

### Eye blink rate (EBR)

A BioSemi ActiveTwo system (BioSemi Inc., Amsterdam) was used to record the EBR. We recorded with two horizontal (one left, one right) and two vertical (one upper, one lower of left eye) Ag-AgCl electrodes, for 6 min eyes-open segments under resting conditions. The vertical electrooculogram (EOG), which recorded the voltage difference between two electrodes placed above and below the left eye, was used to detect eye blinks. The horizontal EOG, which recorded the voltage difference between electrodes placed lateral to the external canthi, was used to measure horizontal eye movements. As spontaneous EBR is stable during daytime but increases in the evening (around 8:30 pm, see Babarto et al., 2000), we never registered after 5 pm. We also asked participants to avoid smoking before the recording. Participants were comfortably sitting in front of a blank poster with a cross in the center, located about 1 m from the participant. The participant was alone in the room and asked to look at the cross in a relaxed state to record EBR1. After mood induction (either positive or negative) EBR2 was recorded. The individual EBR was calculated by dividing the total number of eye blinks during the 6 min measurement interval by 6.

### Mood induction

We used the common mental-imagination procedure (e.g., Strack et al., 1985; Bodenhausen et al., 1994; Phillips et al., 2002; DeSteno et al., 2004; Baas et al., 2008) to induce positive and negative mood. Participants were asked to write down a couple of sentences about an event of their life that made them happy (in a calm, relaxed way) or sad (in a calm, non-angry way), respectively, for 5 min. Calmness was emphasized to keep the two emotional states comparable regarding activation and arousal. EBR2 was recorded right after the mood induction; participants were asked to stop writing but to keep thinking about the event during the measurement interval. The session was completed by filling in the MI2 and the AUT2.

## Results

Before assessing our three experimental hypotheses, we tested whether the experimental groups were comparable before the different moods were induced (see *Comparability of groups*), whether the mood manipulation actually worked (see *Manipulation check*), and whether performance in the creativity task related to individual EBR like it did in the study of Akbari Chermahini and Hommel (2010) [see *Replication of* Akbari Chermahini and Hommel (2010)]. All reported *p* values are for two-tailed testing unless indicated otherwise (one-tailed tests were used for predicted correlations).

### Comparability of groups

A set of independent *t*-test were conducted to check whether the two experimental groups were comparable before undergoing the mood induction. There was not any hint to any pre-experimental difference between the two groups with respect to either the positive or negative subscale of PANAS, and the hedonic valence scores computed from the MI1, nor did any of these scales correlate with EBR1, all *p*s > 0.05. Table 1 provides the relevant information about the mood states in two experimental groups and the four subgroups. Interestingly, the lack of a correlation between EBR1 and pre-experimental mood suggests that mood does not depend on the tonic dopamine level but, if anything, on phasic changes. There was also no hint to a pre-experimental group difference with regard to pre-experimental EBR1 (*p* = 0.14) or flexibility (*p* = 0.88).

**Table 1:** **Means and standard deviations for pre-experimental general mood states (PANAS: positive and negative scales), and current mood states (only hedonic valence score) before (MI1) and after (MI2) mood induction in the two experimental groups, and four subgroups, as a function of low vs. (relatively) high pre-experimental eye blink rate (EBR)**.

**State mood index**		**Mood induction groups**					
		**Positive**			**Negative**		
		**Total (*n* = 43)**	**Low EBR (*n* = 21)**	**High EBR (*n* = 22)**	**Total (*n* = 38)**	**Low EBR (*n* = 19)**	**High EBR (*n* = 19)**
PANAS–PA	M	34.1	33.1	35.1	34.1	33.2	35.1
	S.D.	4.5	4.9	3.9	5.5	4.6	6.1
PANAS-NA	M	16.1	16.2	16.4	16.2	16.4	16.1
	S.D.	4.8	4.9	4.9	6.1	7	5.4
MI1	M	18.1	17.5	18.6	19.9	18.4	20.8
	S.D.	3.1	2.6	3.5	4.0	4.6	3.2
MI2	M	20.9	20.4	21.6	13.4	13.0	13.7
	S.D.	3.1	2.9	3.1	4.7	4.3	5.2

Note: PANAS-PA, PANAS positive affect subscale; PANAS-NA, PANAS negative affect subscale.

### Manipulation check

Another set of paired sample *t*-tests on the hedonic valence score in MI1 and MI2 served to check whether the mood manipulation worked. As expected, participants were significantly more happy after positive-mood induction than before (*M* = 20.95 vs. 18.11), *t*_(42)_ = 5.74, *p* < 0.001,η^2^ = 0.44, and significantly less happy after negative mood induction (*M* = 13.07 vs. 19.65), *t*_(37)_ = 7.76, *p* < 0.001. η^2^ = 0.62. This suggests that the mental-imagery procedure was effective in inducing the respective mood states.

### Replication of Akbari Chermahini and Hommel (2010)

**Figure 2:**
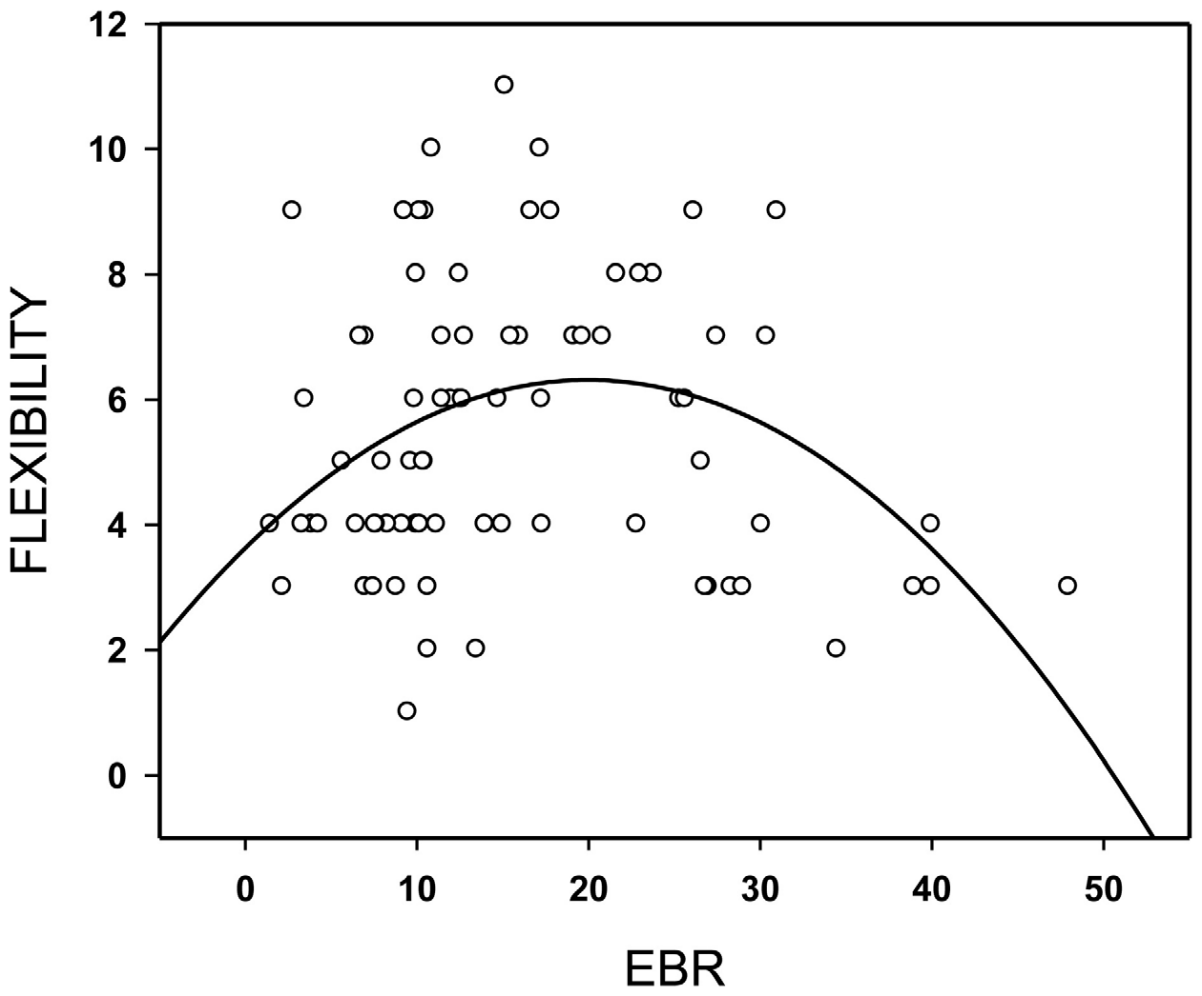
**Flexibility in the divergent thinking task as a function of spontaneous eye blink rate (EBR) per min.** Regression line for best (quadratic) fit.

The relationship between flexibility in the divergent thinking task (AUT1) and EBR1 followed an inverted U-shaped function (Figure 2, quadratic fit = 0.36, *p* = 0.005), whereas the linear fit was poor (0.06, *p* = 0.62)—a pattern that replicates our previous observation (Akbari Chermahini and Hommel, 2010). As in our previous study, there was no significant relation between EBR and one of the other scores of divergent thinking.

### Hypothesis 1: are induced positive (or negative) mood changes reflected in corresponding increases (and decreases) in EBR?

Paired sample *t*-tests revealed systematic changes in EBR after mood induction: As expected, the induction of positive mood led to a significant increase in EBR (*M* = 18.79 vs. 14.1), *t*_(42)_ = 3.8, *p* < 0.001, η^2^ = 0.26. Negative mood induction reduced EBR numerically (*M* = 16.78 vs. 17.39) but this effect was far from significance, *t*_(37)_ = 0.64, *p* = 0.53, η^2^ = 0.01. To summarize, positive-going mood changes are systematically reflected in corresponding EBR changes, while negative-going mood changes are not.

### Hypothesis 2: are positive-going (negative-going) mood and increased (reduced) EBRs associated with increased (reduced) flexibility?

Paired sample *t*-tests assessed the impact of mood induction on performance in the creativity task by comparing flexibility scores before and after the mood manipulation. As expected, the induction of positive mood enhanced flexibility (*M* = 7.1 vs. 5.7), *t*_(42)_ = 3.26, *p* = 0.002, η^2^ = 0.20. The induction of negative mood reduced flexibility numerically (*M* = 5.26 vs. 5.52), but this effect was far from significance, *t*_(37)_ = 0.84, *p* = 0.41, η^2^ = 0.02.

Overall, the correlation between change in cognitive flexibility (AUT2-AUT1) and change in mood (MI2-MI: hedonic valence) was positive and reliable, *r* = 0.24, *p* < 0.018, one-tailed. However, separate analyses revealed that the correlation was positive and pronounced in the positive mood induction group, *r* = 0.44, *p* < 0.001, one-tailed, but negative and unreliable in the negative mood induction group, *r* = −0.18, *p* = 0.28.

**Figure 3:**
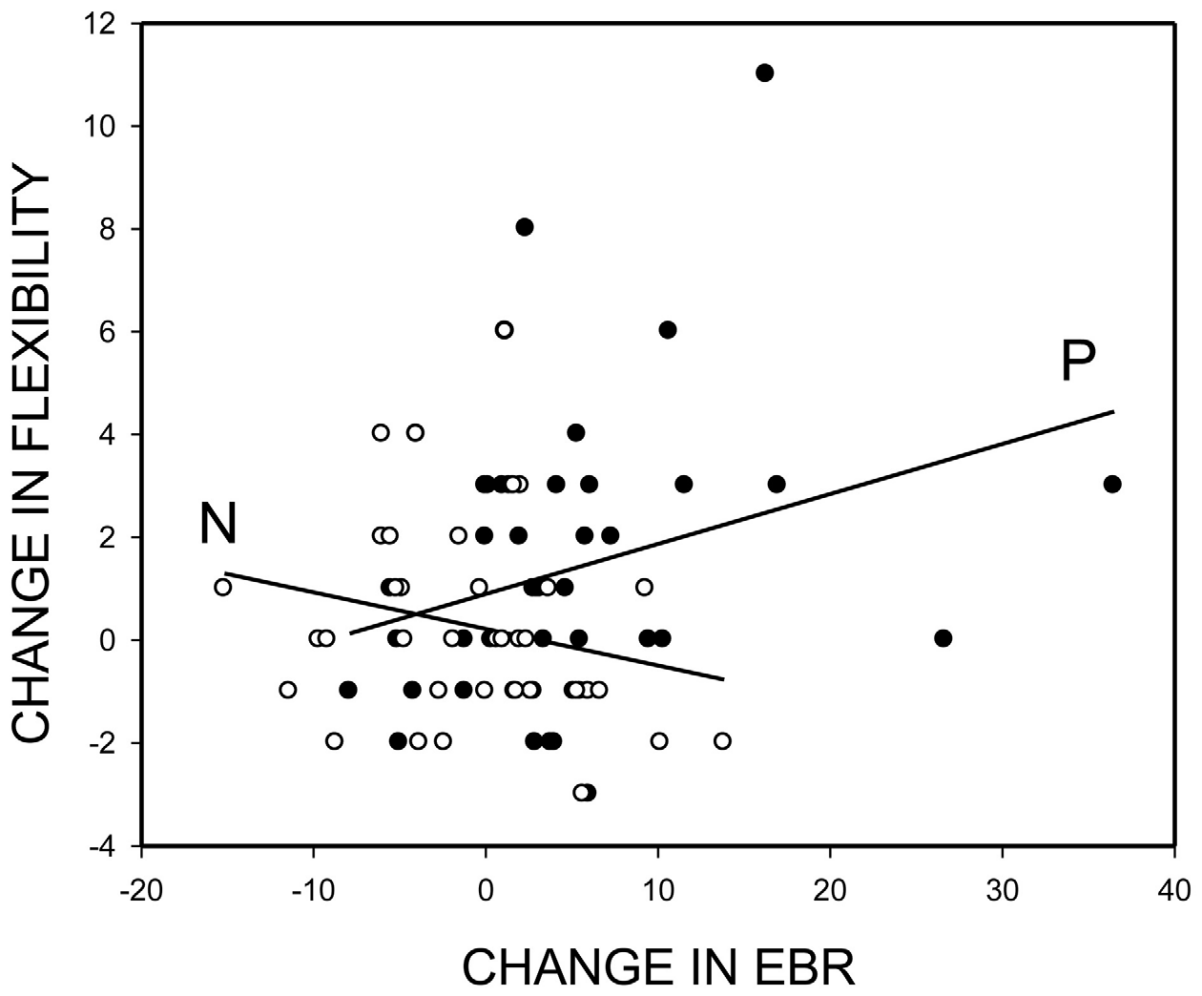
**Mood-induced change in divergent thinking performance (flexibility score post minus flexibility score pre mood induction) as a function of the mood-induced change in spontaneous eye blink rate (EBR).** Empty circles and regression line N for participants with negative-mood induction; filled circles and regression line P for participants with positive-mood induction.

Correlations between change in EBR (EBR2-EBR1) and change in flexibility (AUT2-AUT1: flexibility score) showed a similar pattern. Overall, the correlation was positive and reliable, *r* = 0.19, *p* = 0.047, one-tailed. Separate analyses of the two mood induction groups showed that individuals were becoming more flexible in divergent thinking to the degree that positive mood induction increased their EBR, *r* = 0.29, *p* = 0.03, one-tailed (Figure 3, line: P)—the pattern we expected. In contrast, however, participants in the negative mood group tended to become *more* (rather than less) creative to the degree that negative mood induction decreased their EBR, *r* = −0.23, *p* = 0.17 (Figure 3, line: N)—a pattern that we did not expect.

To summarize, the changes in EBR induced by positive mood induction were systematically related to changes in cognitive flexibility. Although the negative mood induction produced (negative) mood shifts of even greater magnitude, it did not cause significant changes in either EBR or cognitive flexibility. Also, changes in EBR and cognitive flexibility as well as changes in mood and cognitive flexibility were unrelated in the negative mood induction group.

### Hypothesis 3: do individuals with low pre-experimental EBR benefit more (in terms of flexibility) from positive mood more than individuals with higher EBR do?

**Figure 4:**
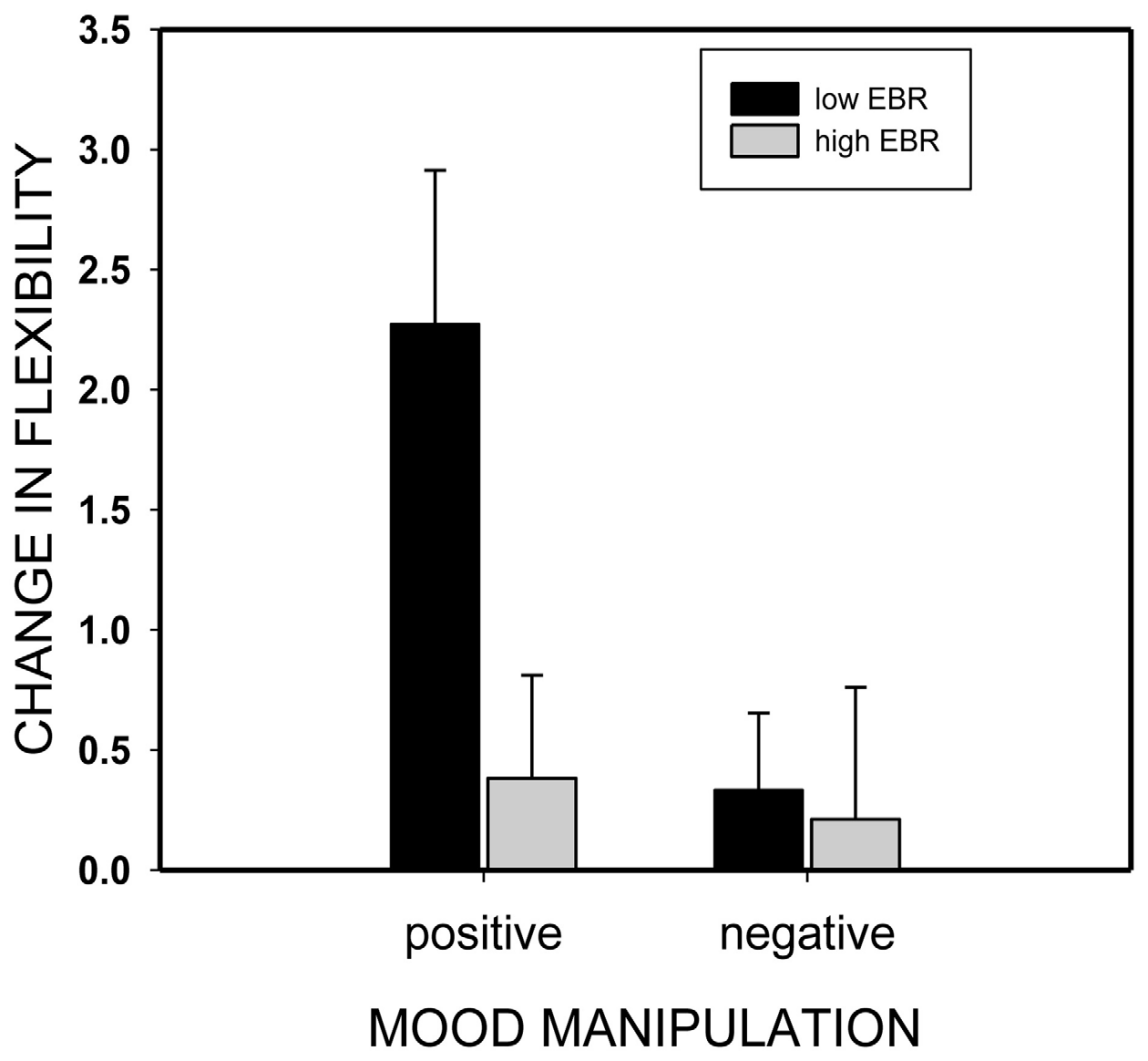
**Change in divergent thinking performance (flexibility score post minus flexibility score pre mood induction) as a function of mood induction (positive or negative) and individual eye blink rate (EBR) level (low or high)**.

We assessed this hypothesis by categorizing participants according to their pre-experimental EBR (EBR1): participants with EBRs below the median were considered low-EBR individuals while participants with EBRs above the median were considered (relatively) high-EBR individuals (which actually represent median-EBR individuals^2^). As expected, and shown in Figure 4, the induction of positive mood improved flexibility only in low-EBR individuals (from 5.8 to 8.0 categories, *t*_(21)_ = 3.54, *p* = 0.002, η^2^ = 0.37) but not in high-EBR participants (5.7 vs. 6.1), *t*_(20)_ = 0.87, *p* = 0.4).

## Discussion

The aim of the present study was to investigate the relationship between mood, flexibility in divergent thinking, and EBR—a marker of individual dopamine levels. Importantly, we were able to fully replicate the inverted U-shaped function relating flexibility to pre-experimental EBR, first reported by Akbari Chermahini and Hommel (2010), which reinforces the notion that flexibility relates to individual dopamine levels.

Our first hypothesis assumes that mood changes are reflected in corresponding changes of the EBR: positive-going mood should increase EBR while negative mood might either reduce EBR or leave it unaffected. Mood changes and EBR changes were indeed correlated and positive clearly increased EBRs; negative mood, in turn, had no reliable impact. This suggests that EBR is a sensitive measure of (some of) the neural processes underlying (positive) mood changes, presumably changes in the individual dopamine level. Even though the functional connection between dopaminergic activity and EBR is not yet well understood and even though the exact quantitative relationship between dopamine level and EBR is not yet known, the finding of a reliable correlation between mood and EBR changes has substantial methodological implications. At the moment, not many ways to assess the current dopamine level of individuals are available: Apart from EBR, dopaminergic activity can be assessed by means of Positron Emission Tomography (Volkow et al., 1996), a rather invasive method, and the advent of high-field MRI may make it possible to scan the current activity level of dopamine-producing nuclei. Hence, in comparison, measuring EBR is a relatively simple, cheap, and non-invasive method that provides at least some insight into dopaminergic activity.

Our second hypothesis assumes that experimentally-induced changes in perceived mood and EBR predict corresponding changes in the flexibility of divergent thinking. As expected, flexibility was improved through the induction of positive mood but not reliable affected by the induction of negative mood. Moreover, the degree of this improvement was predicted by the individual degree to which the mood induction manipulation was successful. Likewise, EBR increased through the induction of positive mood but was not reliable affected by negative mood induction. Finally, the experimentally-induced positive changes in EBR reliably predicted the increase of flexibility. If we assume that EBR reflects changes in dopaminergic activity, this suggests that cognitive flexibility is systematically affected, and perhaps even driven by phasic changes in dopamine. In any case, mood, EBR, and flexibility are related to each other exactly as predicted from dopamine-based approaches to creativity. Interestingly, the predicted relationship was found for the impact of positive mood only, but not for negative mood effects, which provides support for the notion that the functional (e.g., Baas et al., 2008) and neuromodulatory (e.g., Cools et al., 2008; Dayan and Huys, 2008) mechanisms underlying positive and negative mood are different.

According to our third hypothesis, this interrelationship—in view of the fully replicated inverted U-shaped relationship between EBR and flexibility—suggests that individuals with low tonic dopamine levels might benefit more from the induction of positive mood than individuals with medium or high levels do. Indeed, mood-induced improvement of flexibility was only observed in individuals with a pre-experimentally low EBR and a presumably corresponding low tonic dopamine level. Not only does this fit with the non-linear relation between EBR in flexibility reported by Akbari Chermahini and Hommel (2010), it is also likely to explain why unreliable findings and failures to replicate are still abundant in studies on the connection between mood and creativity (Baas et al., 2008; Davis, 2009). Indeed, depending on the particular characteristics and the corresponding distribution of individual dopamine levels in a given sample, the exact same mood-related manipulation can produce significant effects or null results alike, especially if the sample size is small.

Taken together, our findings support the assumption that phasic changes in dopamine levels might provide the common currency underlying the relationship between mood and creativity, as suggested by Ashby et al. (1999) and others, and they provide the hitherto most direct evidence for the underlying interrelationship between mood, creativity, and dopamine. In particular, our findings suggest that elevated mood indeed increases the individual dopamine level and improves aspects of human creativity, as assessed by the flexibility score in our divergent thinking task. At the same time, however, we were able to demonstrate that the reliability and, presumably, the direction of the impact of mood and associated phasic dopamine changes depend on the individual tonic dopamine level (but not the basic mood level!). This questions the generality of claims regarding the positive impact of mood on creativity and calls for closer consideration of individual differences. As our findings show, better mood may or may not facilitate (and may in some cases even impair) creative performance of a given individual. Depending on the specific characteristics of a given sample, this complication may well conceal the true connections between creativity, mood, and dopaminergic activity in empirical studies and applied settings.

In the light of our findings, a number of further questions present themselves. For instance, it remains to be seen whether a comparable interrelationship exists between mood, dopamine, and convergent thinking—which apparently relates to tonic dopamine levels in different, and in some sense opposite, ways than divergent thinking does (Akbari Chermahini and Hommel, 2010). Recently we observed that engaging in convergent thinking leads to more negative mood (Akbari Chermahini and Hommel, 2011), which would fit with this expectation. Moreover, it seems important to clarify the functional relationship between mood and phasic dopaminergic changes. After all, mood is a concept that relates to a personal level of description and relates to a person having and experiencing it. In contrast, changes in dopaminergic activity refer to the systems level of description, which may or may not correspond to personal-level concepts in a one-to-one fashion. Hence, it would be important to understand whether and to what degree dopaminergic changes are the neural reflection of being in a particular mood, or whether they are mere by-products of particular mood states.

### Conflict of interest statement

The authors declare that the research was conducted in the absence of any commercial or financial relationships that could be construed as a potential conflict of interest.

## Footnotes

^1^Akbari Chermahini and Hommel (2010) assessed divergent thinking by means of Guilford (1967) Alternate Uses Task, which was also used in the present study. Responses in this task are commonly scored according to flexibility, originality, fluency, and elaboration, to quantify the number of different categories used, the uniqueness and number of responses, and the amount of detail, respectively. As Akbari Chermahini and Hommel (2010) found systematic, reliable findings for flexibility only (an observation that we replicated in the present study), and because most theoretical claims relate to flexibility (e.g., Ashby et al., 1999; Hommel, 2012), the present study was focusing on this variable.

^2^Informal observations from our lab revealed that people with very high EBR levels are rare in our student population and more often than not report to have family members with schizophrenia. This fits with the distribution of EBRs in Akbari Chermahini and Hommel (2010) and in the present study, where the EBRs of the majority of participants falls on the left, ascending part of the inverted U-shaped function relating EBR to divergent thinking. If we in the following distinguish between below- and above-median EBRs, it should therefore be kept in mind that even above-median EBRs in the present study are actually representing medium EBRs in the general population. In other words, even though we will compare individuals with low vs. high EBRs, the present study actually compares individuals with low vs. medium EBRs.
